# Supplementary material for: Consumption of Lactose, Other FODMAPs and Diarrhoea during Adjuvant 5-Fluorouracil Chemotherapy for Colorectal Cancer
Source: Nutrients. 2020 Feb 4;12(2):407. doi: 10.3390/nu12020407 (PMC7071323; doi:10.3390/nu12020407)
Supplement: Supplementary file 1 [file nutrients-12-00407-s001.pdf]

Supplements

Figure S1. Assessment chart.

			Cycle 1				Cycle 2				Cycle 3				
Chemotherapy	Performed	Baseline -1 week	Week 1	Week 2	Week 3	Week 4	Week 5	Week 6	Week 7	Week 8	Week 9	Week 10	Week 11	Week 12	Week 13
Mayo	50 %		↓↓↓↓↓				↓↓↓↓↓				↓↓↓↓↓				↓↓↓↓↓
sLV5FU2	50 %		↓→		↓→		↓→		↓→		↓→		↓→		
Postoperative chemoradiation	27 %										→→→→→→→→→→→→→→→→→→→→→→→→→→→→→→				
Dietary supplementation															
Lactobacillus rhamnosus	63 %		→→-												
Guar gum fiber (8 days/cycle)	29 %			XXXX		XXXX		XXXX		XXXX		XXXX		XXXX	
Assessments															
Lactose tolerance test	94 %	X												X	
Methane test	85 %	X													
Toxicity diary and grading	100 %		Worst grade toxicity								Worst grade toxicity				
Food diary	100 %	XXXX												XXXX	

Table S1. Comparison of the baseline characteristics in patients with different combinations of lactose-rich and FODMAP-rich foods during cycles I and III. 'None' indicates low lactose and low FODMAP, 'L or F' indicates high lactose or high FODMAP and 'L and F' indicates high lactose and high FODMAP.

		Cycle I							Cycle III						
		None		L or F		L and F			None		L or F		L and F		
		N	%	N	%	N	%	P*	N	%	N	%	N	%	P*
Gender	Male	5	35.7	12	54.5	9	56.3	0.46	7	46.7	10	45.5	9	60.0	0.65
	Female	9	64.3	10	45.5	7	43.8		8	53.3	12	54.5	6	40.0	
Lactase deficiency status	Normolactasia	7	58.3	16	72.7	12	80.0	0.36	9	69.2	13	61.9	13	86.7	0.46
	Borderline	3	25.0	1	4.5	1	6.7		2	15.4	3	14.3	0	0.0	
	Hypolactasia	2	16.7	5	22.7	2	13.3		2	15.4	5	23.8	2	13.3	
Methane status producer	Non-producer	10	76.9	9	52.9	7	50.0	0.29	7	53.8	10	58.8	9	64.3	0.86
	Producer	3	23.1	8	47.1	7	50.0		6	46.2	7	41.2	5	35.7	
Cancer stage	Stage II	2	14.3	5	22.7	2	12.5	0.73	3	20.0	4	18.2	2	13.3	0.52
	Stage III	11	78.6	13	59.1	12	75.0		11	73.3	13	59.1	12	80.0	
	Stage IV	1	7.1	4	18.2	2	12.5		1	6.7	5	22.7	1	6.7	
Site	Colon	9	64.3	12	54.5	11	68.8	0.65	11	73.3	12	54.5	9	60.0	0.51
	Rectum	5	35.7	10	45.5	5	31.3		4	26.7	10	45.5	6	40.0	
Type of surgery	Right hemicolectomy	2	14.3	5	22.7	6	37.5	0.30	5	33.3	5	22.7	3	20.0	0.92
	Left hemicolectomy or sigma resection	4	28.6	6	27.3	5	31.3		5	33.3	5	22.7	5	33.3	
	Rectum resection	5	35.7	10	45.5	5	31.3		4	26.7	10	45.5	6	40.0	
	Subtotal colectomy	3	21.4	1	4.5	0	0.0		1	6.7	2	9.1	1	6.7	
Abdominal stoma	No	8	57.1	16	72.7	12	75.0	0.51	12	80.0	14	63.6	10	66.7	0.55
	Yes	6	42.9	6	27.3	4	25.0		3	20.0	8	36.4	5	33.3	
Chemotherapy	Mayo regimen	7	50.0	14	63.6	5	31.3	0.14	7	46.7	12	54.5	7	46.7	0.85
	Simplified de Gramont regimen	7	50.0	8	36.4	11	68.8		8	53.3	10	45.5	8	53.3	
Radiotherapy	No	11	78.6	14	63.6	13	81.3	0.42	12	80.0	16	72.7	10	66.7	0.71
	Yes	3	21.4	8	36.4	3	18.8		3	20.0	6	27.3	5	33.3	
Type of supplementation	None	7	50.0	10	45.5	2	12.5	0.19	4	26.7	11	50.0	4	26.7	0.45
	LGG® only	4	28.6	7	31.8	7	43.8		6	40.0	7	31.8	5	33.3	
	LGG®+fibre	3	21.4	5	22.7	7	43.8		5	33.3	4	18.2	6	40.0	

*) Chi-squared test
